# Antimicrobial Activity of Zinc against Periodontal Pathogens: A Systematic Review of In Vitro Studies

**DOI:** 10.3390/medicina59122088

**Published:** 2023-11-28

**Authors:** Viktorija Griauzdyte, Egle Jagelaviciene

**Affiliations:** 1 UAB Vilnius Implantology Center Clinic, A. Vivulskio Str. 7-102, LT-03162 Vilnius, Lithuania; viktorija.griauzdyte@gmail.com; 2 Department of Dental and Oral Pathology, Lithuanian University of Health Sciences, Eiveniu Str. 2, LT-50161 Kaunas, Lithuania

**Keywords:** zinc, zinc oxide, periodontal diseases, periodontitis, *Porphyromonas gingivalis*

## Abstract

*Background and Objectives*: More than a billion people worldwide suffer from chronic periodontitis. The primary etiological factor of periodontal diseases is dental plaque and the bacteria it contains, particularly *Porphyromonas gingivalis*, *Tannerella forsythia*, *Treponema denticola*, *Prevotella intermedia*, and *Aggregatibacter actinomycetemcomitans*. Zinc, owing to its antibacterial properties, can be employed in periodontology. The objective of this review was to analyze scientific literature that examines the effects of zinc on periopathogens. *Materials and methods*: A systematic review protocol of scientific literature was designed following PRISMA recommendations. Data search was conducted in PubMed, Web of Science, and ScienceDirect databases. Full-text articles in English that examine the effects of zinc on periopathogens and were published between 2011 and 2021 were included. *Results:* Fifteen articles were included in the analysis based on inclusion criteria. ZnO exhibited antibacterial activity against *P. gingivalis* and *P. intermedia* (*p* < 0.001). The minimum inhibitory concentration against *P. gingivalis* was 10 μg/mL. ZnO demonstrated a significant antibacterial effect, as evidenced by inhibition zones of 15.10 mm for *S. oralis*, 13.36 mm for *P. gingivalis*, 12.98 mm for *S. sanguis*, and 14.01 mm for *P. intermedia.* Zn (II)-based polymers inhibited the *ragA* and *ragB* genes of *P. gingivalis*. Titanium dental implants coated with ZnO effectively disrupted the cell walls of *P. gingivalis* and *A. actinomycetemcomitans*. ZnO inhibited the growth of *P. gingivalis* within 2 h and the growth of *F. nucleatum* and *P. intermedia* within 3 h. ZnO exhibited nontoxic effects, and concentrations up to 0.8 mg/L increased cell survival rates by up to 90%. *Conclusions:* The analysis of the literature confirms the antibacterial action of zinc against periodontal pathogenic bacteria. At low concentrations, these substances do not exhibit cytotoxic effects on fibroblasts.

## 1. Introduction

Zinc is a metal found in the Earth‘s crust. It is an irreplaceable and essential element for human health. Approximately 60% of zinc is located in muscles; 30% in bones; and the remaining 10% in skin, hair, pancreas, kidneys, and blood plasma [1]. Intracellularly, it is found in the cytoplasm, vacuoles, organelles, and nucleus [2]. As one of the essential elements and the second most widely distributed chemical element in the human body after iron, it supports the optimal functions of organisms by catalyzing enzyme activity, contributing to the protein structure, and regulating gene expression [3]. Zinc plays a vital role in immune reactions and both cellular and humoral immunity [2]. This trace element is necessary for human health, and its deficiency can lead to various diseases. Zinc is also present in the oral cavity, found in saliva, dental plaque, and enamel hydroxyapatites [4].

The onset and development of periodontal diseases are related to many risk factors as this is a multifactorial pathology, including the inflammatory–immune response of the organism and the metabolism of connective and bone tissues. The etiology of these diseases includes local risk factors (soft and mineralized plaque, dental caries, and iatrogenic factors), systemic diseases, age, sex, heredity, poor oral hygiene, smoking, medication use, and stress. Periodontal diseases have an infectious origin and are associated with a mixed bacterial flora. Various types of microorganisms and bacteria colonize the oral cavity, interacting with each other and with cells and tissues, thereby influencing physiological processes and causing pathological conditions. Examination of the microbiology of periodontal diseases and their inflammatory systems suggests that the inflammatory response and the periodontal microbiome represent a bidirectional balance in oral health and that there is a bidirectional imbalance in periodontal diseases [5]. There is no doubt that dysbiosis is involved in the development of periodontal disease, but whether dysbiosis itself causes the disease or whether the disease causes dysbiosis is still under investigation. Additionally, the importance of individual bacterial species in the development of dysbiosis has been proven; one of them is *P. gingivalis*, which will be examined in this work. According to recent findings, the changes in colonizing bacteria associated with the pathological process leads to significant alterations in the overall structure and functional properties of the microbial population [5].

Periodontal diseases develop when the pH in the mouth changes and pathogenic bacteria start to multiply and colonize, causing them to secrete metabolites. When pathogens interact with periodontal tissues, specific and nonspecific immune responses develop, which either stop without consequences or damage the periodontal tissues as they progress. The severity and outcome of the pathological process in the periodontium depend on the virulence and pathogenicity of the microorganism and the expression of the host immune response, which can be individual and depend on distinct factors. A limited group of bacteria plays a key role in regulating the progression of clinical signs associated with periodontal diseases, such as gingival inflammation (gingivitis) accompanied by redness of the gingiva, swelling, pain, and bleeding, which, if left untreated, becomes periodontitis with periodontal pocket formation, attachment, and bone loss [6]. Bacterial metabolites damage the periodontal tissues, and inflammation-related tissue loss is the primary cause of tooth loss. Gingivitis or periodontitis, with varying degrees of severity, affects more than 70% of adults worldwide [7].

The purpose of periodontal therapy is to suppress the activity of bacterial microflora, reduce or eliminate subgingival infection, prevent the formation of periodontal pockets, and prevent disease recurrence [8]. One of the treatment methods involves scaling mineralized supra- and subgingival dental plaque and root planing. Combining routine scaling of mineralized plaque with the use of antimicrobial substances is considered the most effective approach for improving periodontal tissue condition and reducing periodontal pathogens as it helps mitigate the progression of periodontal diseases [9]. Antimicrobial agents can act both systemically and topically with controlled release. Zinc (Zn) is an antimicrobial substance that has been used in medicine for several decades [8]. Research has demonstrated the antimicrobial efficacy of zinc oxide nanoparticles [10,11]. The application of zinc in periodontology has not been thoroughly investigated yet. Scientists are still studying the effects of zinc on oral bacteria and inflammatory periodontal tissues. The results should help answer whether the topical application of zinc (as part of a medication system) can accelerate or enhance the healing of periodontal tissues as this substance can be released directly to the target location at constant concentrations [4,8,9,10,11,12,13,14,15,16,17,18,19,20,21]. Topical administration of zinc medications into periodontal tissues could provide prolonged action and effectiveness [9]. This systematic review addresses the following question: “Does zinc affect bacteria associated with periodontal disease”? The aim of the study was to examine the effects of zinc and zinc compounds on the vital activity of periodontal pathogens and their cytotoxicity on periodontal tissue cells.

## 2. Material and Methods

### 2.1. Protocol and Registration

The systematic review of scientific literature was approved by the Center of Bioethics of Lithuanian University of Health Sciences (Permission N, BEC-OF-92, 2022-03-24). The PRISMA (Preferred Reporting Item for Systematic Review and Meta-Analyses) protocol was used to plan this systematic review of scientific literature, formulate the study aim, select scientific publications, assess test relevance, and analyze the selected data [22].

### 2.2. Search Strategy and Data Source

The search of scientific publications in PubMed, Web of Science, and ScienceDirect databases was conducted from 5 May 2021 to 15 September 2021. Abstracts, publications, and their references that analyzed bacteria causing periodontal diseases and in vitro studies on the effects of zinc on these bacteria were selected and reviewed. Keywords and their combinations were utilized for the search, and additional filters were activated in Web of Science and ScienceDirect electronic databases. Before reaching a final decision, two reviewers assessed publication titles and abstracts, and the full text of each selected study was then evaluated. Disagreements among the reviewers were discussed. The results of the publication search are presented in Table 1.

### 2.3. Eligibility Criteria

Full-text articles in English published between 2011 and 2021 and examining the effects of zinc on periopathogens in vitro as well as its cytotoxic effect on periodontal tissues cells were selected, and a systematic scientific review was conducted. Systematic scientific reviews, theses, books, clinical trials involving patients, and articles not in English or published before 2011 were excluded.

### 2.4. Data Collection

**Figure 1:**
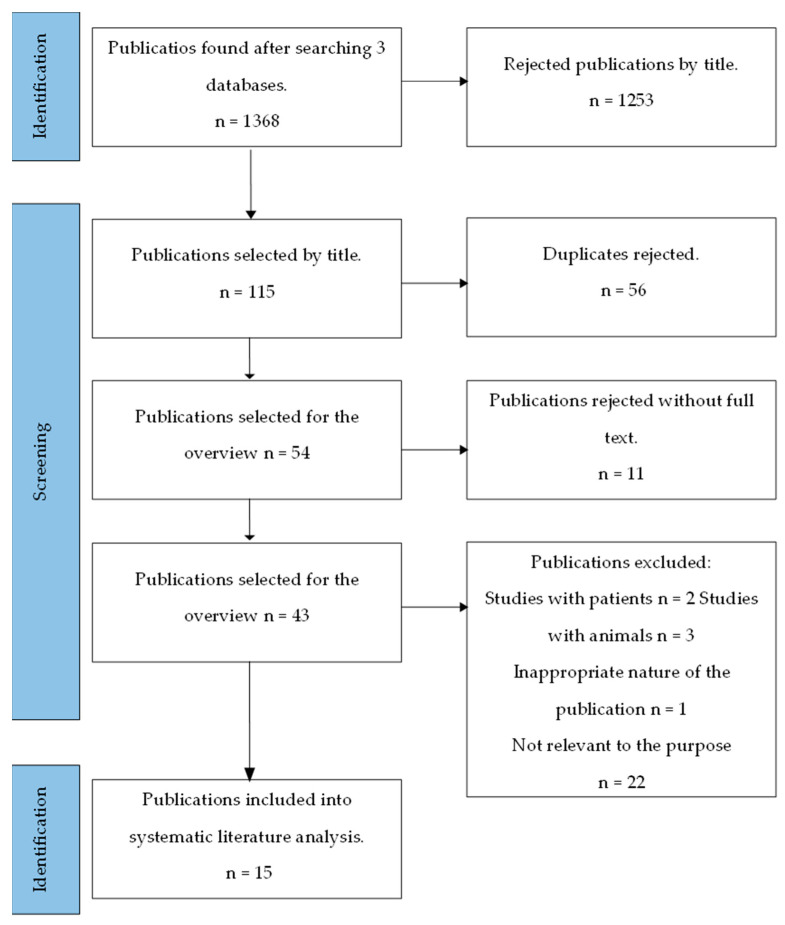
Diagram of PRISMA-based strategy of publication search [22].

Data from the selected articles were recorded in a flow chart that specified the main author, year of publication, study types, types of bacteria (*Porphyromonas gingivalis*, *Tannerella forsythia*, *Treponema denticola*, *Prevotella intermedia*, and *Aggregatibacter actinomycetemcomitans)*, and the methodologies used in the studies. The flow chart was used to analyze both the bactericidal and bacteriostatic effects of zinc on bacteria, and its cytotoxic effects on gingival fibroblasts were analyzed. The results of the study are presented herein. The PRISMA flow diagram and detailed article search strategy is shown in Figure 1.

### 2.5. Risk of Bias Assessment

The OHAT risk of bias assessment tool was used to evaluate systematic errors of the selected publications [23]. The risk assessment of systematic errors in in vitro studies was based on evaluation algorithms, which categorized risk into four levels ranging from very low to high. It was determined that all 15 studies were at a low risk level of systematic errors. The following aspects were analyzed: random selection of dose or exposure levels, concealed allocation to groups, consistent study conditions, independence of examiners, comprehensiveness of outcome data (without deletion or exclusion from analysis), appropriateness of effect characterization, confidence in outcome assessment, reporting of all obtained results, and the ultimate outcome.

## 3. Results

### 3.1. General Characteristic

Performing a publication search with filters, 1368 articles were found. Out of these, 115 articles with keywords in the titles were selected. Finally, 15 articles were chosen based on inclusion and exclusion criteria. All the scientific publications examined the in vitro antibacterial activity of zinc-ion-containing substances on at least one bacterium associated with periodontal diseases [4,8,9,10,11,12,13,14,15,16,17,18,19,20,21], while four publications investigated the toxicity of Zn compounds on gingival fibroblasts [4,8,10,12]. The activity of zinc against bacteria was studied during 24 and 48 h periods, with the longest test duration being three days [20]. Table 2 provides a general overview of the examined publications, and Table 3 presents an overview of the tested bacteria and their antibacterial activity based on bacterial inhibition. During analysis, particular attention was given to the main pathogenic bacteria: 15 publications studied *P. gingivalis*, the primary periopathogen; 6 publications focused on *P. intermedia* [4,12,13,14,15,16] and *A. actinomycetemcomitans* [4,13,14,15,16,20], and 1 publication investigated *T. forsythia* and *T. denticola* [4].

Antibacterial activity was tested using different substances in various concentrations. ZnO nanoparticles were the primary test substance in four publications [8,10,16,18], while 11 publications also examined an additional zinc-containing compound [4,9,11,12,13,14,15,17,19,20,21]. In one publication, free ZnO was studied in combination with an additional substance, polycaprolactone [4]. Polycaprolactone nanofiber is a biomaterial that mimics the natural intercellular matrix. It is a semicrystalline aliphatic polyester characterized by biodegradability, high biocompatibility, thermal stability, and good mechanical properties. These properties make polycaprolactone nanofibers a suitable choice for integration with the test materials [4]. Mou et al. developed an albumin-based medication system with enhanced stability of albumin nanoparticles and antibacterial activity after the addition of ZnO [12]. Two pairs of investigators examined each material. Frober et al. and Bergs et al. studied zinc in combination with glucose-1-phosphate [13,14]. In subsequent studies, scientists combined nanoparticles of zinc peroxide with a biofunctionalized stabilizer of glucose-1-phosphate to enhance the material’s interaction with bacterial walls in a more specific manner [13]. They also synthesized zinc peroxide together with bioactive nanomolecules of sugar and assessed the release of oxygen and antibacterial activity [14]. Additionally, researchers have analyzed a material supporting bivalent zinc (Zn(NO_3_)_2_·6H_2_O) [11,21], synthesized a polymer supporting bivalent zinc [Zn(TBTA) (L)1.5]n [17], modified the surfaces of titanium implants with zinc [20], and synthesized dextran-coated zinc hydroxyapatite. Dextran has natural resistance to salivary microorganisms, and dextranase acts on plaque bacteria. It was noted that the surface area, reactivity, and biomimetic morphology of hydroxyapatite nanoparticles increased biocompatibility and antibacterial properties [19].

The antibacterial activity of the substance has been assessed using various methods. Three publications [4,8,12] measured inhibition zones caused by the substance, with the size of the inhibition area indicating the absence of bacterial growth, measured in millimeters. Seven publications evaluated the antibacterial activity of Zn in terms of minimum inhibitory concentration (MIC) and minimum bactericidal concentration (MBC), which are required to halt bacterial growth, multiplication, and survival [8,9,10,13,14,15,16] (refer to Table 4). In these tests, the same quantity of bacteria was exposed to various concentrations of the substance. MIC represents the lowest concentration of the antibacterial agent needed to inhibit further growth of the specific bacteria. MBC is the lowest concentration of the antibacterial agent necessary to kill the specific bacteria. An additional test was required to determine this concentration: bacteria were recultured for a few days after their exposure to the test substance, and the tested concentration of the substance was considered as MBC if there was no bacterial growth. Authors assessed the antibacterial activity of zinc through bacterial growth curves in five publications [8,11,15,17,21]. The calculation of bacterial growth curves is based on optical density absorbance measured by a spectrophotometer at different wavelengths. The absorbance value directly correlates with bacterial concentration in the solution.

The authors measured bacterial absorbance and determined bacterial concentration before exposure to the test substance. These measurements of bacterial absorbance were taken every hour, resulting in the generation of growth curves. Two scientific publications demonstrated the antibacterial activity of zinc through quantitative evaluation of bacterial culture using different staining methods to distinguish viable and dead bacteria [13,20]. A special fluorescent dye was employed to assess the antibacterial activity of the test substance, which stains viable bacteria in green and dead bacteria in red. Results were observed through fluorescent microscopy. Frober et al. focused their study on staining one type of bacteria, *A. actinomycetemcomitans* [13]. Examination of bacterial viability was based on their ability to reduce tetrazolium salt into formazan dye after exposure to zinc [18]. Tetrazolium salt was introduced into bacterial culture, where viable bacteria reduced it into purple formazan dye. Viable bacteria were identified using a spectrophotometer. Antibacterial activity was evaluated through bacterial absorbance [19], where the absorbance value of the bacterial solution depended on the bacterial concentration within it.

The genes *ragA* and *ragB* are known to participate in the process of *P. gingivalis* growth. Therefore, *ragA* and *ragB* may serve as specific indicators of treatment outcomes [17]. In two scientific studies [17,21], the main *P. gingivalis* growth genes and the effect of zinc on them were analyzed using supplementary polymerase chain reactions (PCR). The study also examined the antibacterial activity of the synthetic compound that supports bivalent zinc ([Zn(TBTA) (L)1.5]n), its impact on *P. gingivalis* growth, and the results of PGR test for *ragA* and *ragB* genes [17].

Authors analyzed the cytotoxic activity of zinc on mouse and human gingival fibroblasts in four scientific publications. Cell viability after exposure to the test substance was determined by the cell’s ability to reduce tetrazolium salt into formazan dye, and cell absorbance was examined using a spectrophotometer. Cell viability was expressed as a percentage, calculated based on the proportion of remaining viable cells [4,8,10,12]. Among these studies, three examined zinc cytotoxicity on mouse gingival fibroblasts [4,8,12], while one study focused on the effect of ZnO nanoparticles on human gingival fibroblasts [10].

### 3.2. Characterization of Scientific Studies

One of the studies investigated nanoparticles of polycaprolactone (PCL), oxytetracycline hydrochloride (OTC), and zinc oxide (ZnO) and their applications in periodontology [4]. The authors hypothesized that combining two antibacterial agents against periodontal bacteria and incorporating OTC together with ZnO into PCL nanoparticles could ensure prolonged medication release. They prepared a control substance of only PLC and three test substances of PLC nanoparticles with the addition of OTC, ZnO, and OTC combined with ZnO and examined their antimicrobial activity and cytotoxicity against mouse gingival fibroblasts. Microbiological studies were conducted using the following species of bacteria: *Aggregatibacter actinomycetemcomitans*, *Porphyromonas gingivalis*, *Fusobacterium nucleatum*, and *Prevotella intermedia.* The agar diffusion assay was employed to assess antimicrobial activity against bacterial cultures. Sterile filter paper discs impregnated with OTC, OTC/ZnO, and ZnO were placed on top of the Petri dish. A 70% ethanol solution that exhibited no bacterial inhibition served as the control. Bacterial cultures were incubated anaerobically (Table 3). Assays were repeated three times, and the diameter of the inhibition zones around the discs was measured in millimeters. The inhibition zones of the compound substances PCL-OTC and PCL-OTC/ZnO were found to be greater than those of the single components. Cytotoxicity tests were conducted to evaluate mouse fibroblast viability after 24 and 48 h of exposure to the test samples. The mean percentage of cells viability following exposure to PCL-ZnO was 97.0% and 95.77% after 24 and 48 h, respectively. The results demonstrated the nontoxic nature of the test substance.

The antibacterial properties of ZnO were studied while varying the size and number of nanoparticles [12], and the test sample was prepared by dissolving equal amounts of ZnO nanoparticles, albumin, and minocycline. Antibacterial activity was assessed by measuring the inhibition zone. Bacterial species, including *S. oralis/S. mitis*, *P. gingivalis*, and *S. sanguinis/S. gordonii*, were inoculated on lysogeny agar plates, and the test substance was instilled and incubated for 24 h. The results indicated that the inhibition zones of *S. oralis*, *P. gingivalis*, *S. sanguinis/S. gordonii*, and *P. intermedia* increased with increasing concentrations of ZnO nanoparticles ranging from 0.2 to 0.8 mg/L. This confirmed the antibacterial activity of the test substance. Additionally, during the same study, the cytotoxicity of the test substance on gingival cells was assessed. The cells were incubated with the test substance for 24 h, and cell counts showed that cell viability increased from 25% to 85–90% with the addition of ZnO to free minocycline. In the following study, the antimicrobial effects of ZnO nanoparticles combined with glucose-1-phosphate (ZnO + Glc-1P) were conducted in vitro. The mixture (ZnO + Glc-1P) was used in different ratios throughout the study [13]. The interaction between the test substance and bacteria was visualized through various staining techniques to distinguish between viable and dead cells. The activity of the test substance against a range of bacteria, including *F. nucleatum*, *P. gingivalis*, *P. intermedia*, *A. actinomycetemcomitans*, *E. faecalis*, *L. aureeius*, *S. aureus*, and *C. albicans*, was analyzed. The minimum inhibitory concentration (MIC) and minimum bactericidal concentration (MBC) of nanoparticle combinations were determined using the microtitration method in Petri dishes. Samples were incubated until visible inhibition of bacterial growth occurred. The results indicated that ZnO particles strongly inhibited the growth of all Gram-negative bacterial species, including *A. actinomycetemcomitans*, *P. gingivalis*, *P. intermedia*, and *F. nucleatum*.

One more study was conducted on the effect of ZnO nanoparticles (ZnONP) on bacterial function [8], where the experiments consisted of two stages: in the first stage, the inhibition zone and MIC against *P. gingivalis* and *A. Naeslundii* were determined to assess antibacterial activity; in the second stage, the biocompatibility of ZnONP was evaluated using mouse fibroblasts. The antimicrobial activity of the substance was tested using the microtitration method, and a spectrophotometer with a 600 nm wavelength was employed to measure bacterial concentration. The agar diffusion test was used to evaluate the zone of bacterial growth inhibition. In this test, a Petri dish was inoculated with bacterial cultures, the test substance was added, and the inhibition zone was measured in millimeters (mm) (Table 3). The MIC of ZnONP against *P. gingivalis* was found to be 10 μg/mL, while the MIC against *A. naeslundii* was 40 μg/mL. The viability of mouse fibroblast exceeded 60%, confirming the biocompatibility of ZnONP.

Continuing the scientific literature review analysis, a study of a mixture of zinc peroxide nanoparticles with glucose-1-phosphate (Glc-1P) was analyzed [14]. The study investigated the antibacterial activity of the synthesized nanoparticles against *E. faecalis*, *A. actinomycetemcomitans*, *P. gingivalis*, and *P. intermedia*. The study employed the agar diffusion test, where the test substance was added to inoculate bacterial cultures and the inhibition of bacterial growth observed to establish MIC. The results of the study indicated that *P. intermedia*, *A. actinomycetemcomitans*, and *P. gingivalis* were most susceptible to Zn compounds. The antimicrobial activity of zinc chloride (ZnCl_2_) was also studied [15]. Eight species of oral microorganisms responsible for halitosis and periodontal disease, including *A. actinomycetemcomitans*, *F. nucleatum*, *P. gingivalis*, *P. intermedia*, *T. denticola*, *T. forsythia*, *St. aureus*, *and S. mutans*, were treated with a ZnCl_2_ solution. Growth curves were generated, and the minimum inhibitory concentration (MIC) was expressed as a percentage. The antibacterial activity of ZnCl_2_ was assessed by measuring the optical density of bacterial cultures at a wavelength of 600 nm. The results confirmed the efficacy of the ZnCl_2_ solution in inhibiting bacterial growth. The highest MIC of ZnCl_2_ was observed against *S. mutans* (0.5%) and *A. actinomycetemcomitans* (0.25%), while it was lower against *P. gingivalis*, *P. intermedia*, and *T. denticola* (0.06%).

The antimicrobial activity of nanoparticles from various substances, including silver, copper oxide, dicopper oxide, zinc oxide, titanium oxide, tungsten oxide, a composite compound of silver and copper oxides, and a composite compound of silver and zinc, was investigated [16]. Their effects were determined based on the estimation of MIC and MBC against *P. intermedia*, *P. gingivalis*, *F. nucleatum*, *and A. actinomycetemcomitans.* MIC and MBC values were calculated using the microtitration test, and bacterial concentration was measured by optical density at 540 nm. The activity of the nanoparticles, ranked in descending order, was as follows: silver > composite compound of silver and copper oxide > dicopper oxide > copper oxide > composite compound of silver and zinc oxide > zinc oxide > titanium dioxide > tungsten oxide. Notably, ZnO significantly inhibited bacterial growth, completely inhibiting *P. gingivalis* within two hours and *F. nucleatum* and *P. intermedia* within three hours (Table 3).

The antibacterial activity of a new bivalent-zinc-containing polymer, [Zn(TBTA) (L)1.5]n, against P. gingivalis was studied, and the activity of the synthetic compound was evaluated and *P. gingivalis* growth curves obtained [17]. Bacterial growth was estimated by optical density at 560 nm. The results proved the antibacterial properties of the compound. Real-time polymerase chain reaction was performed on *P. gingivalis ragA* and *ragB* genes. The results proved that the bivalent-zinc-containing polymer could suppress the expression of *ragA* and *ragB* genes, inhibiting the growth of *P. gingivalis* in the periodontium.

The purpose of another study was to synthesize antibacterial nanoparticles filled with calcium and zinc ions or doxycycline [18]. Bacterial species, including *P. gingivalis*, *L. lactis*, *S. mutans*, *S. gordonii*, and *S. sobrinus*, were investigated. For each bacterial species, 1 mL of suspension was incubated with nanoparticles suspended in a phosphate buffer solution at three different concentrations for 3, 12, and 24 h. Bacterial viability was assessed based on their ability to convert tetrazolium salt into formazan dye. Tetrazolium salt (3-(4.5-dimetiltiazol-2-il-)-2.5-difeniltetrazolium bromide) transforms into the chromophore formazan when the reduction of pyridine dinucleotides (NAD(P)H) occurs. The amount of formazan obtained and viable cells are directly proportional. The aqueous solution of tetrazolium salt is yellowish, while the solution of formazan salt is purple. The most effective antibacterial substance was doxycycline nanoparticles (resulting in a reduction of bacterial count by 60% to 99%), followed by nanoparticles of calcium or zinc (resulting in a reduction of bacterial count by 30% to 70%). The most susceptible species were *P. gingivalis*, *S. mutans*, and *L. lacti*, while *S. gordonii* and *S. sobrinus* species exhibited resistance to the tested nanoparticles.

The antimicrobial activity of o-cymen-5-ol and zinc glutamate was evaluated based on their MIC and MBC values [9], encompassing five species of periodontium bacteria: *S. mutans*, *A. viscosus*, *P. gingivalis*, *F. nucleatum*, and *C. Albicans.* These bacteria were incubated with the test substance at different concentrations for 48 h, and bacterial absorbance was measured at a wavelength of 550 nm. The minimum concentration that inhibits bacterial growth is defined as the MIC. Bacteria affected by the test substance were inoculated on blood agar. The MIC values for o-cymeno-5-ol and zinc gluconate were determined to be 0.42 and 0.69 mM against *P. gingivalis* and *F. nucleatum*, respectively, while the MBC values were 0.83 and 1.66 mM, respectively. Zinc demonstrated a greater ability to suppress bacterial glycolysis and protease activity compared to o-cymen-5-ol. The combination of the zinc and o-cymen-5-ol system demonstrates direct antimicrobial activity and inhibits processes related to oral diseases.

Studies have also investigated the antibacterial activity of zinc oxide nanoparticles (ZnONPs) and their cytotoxic effects on human gingival fibroblasts [10] by exposing S. *sanguinis*, *P. gingivalis*, *P. melaninogenica*, and *S. mutans* to ZnONPs and determining the MICs. Viable and dead cells were examined using a laser microscope. The MIC of ZnONP against bacterial cultures ranged from 78.3 to 3906 μg/mL, with the specific concentration values not being distinguished for each bacterium. An MBC of 125 μg/mL was considered significant. The study revealed significant differences in the biomass of viable and dead bacteria after exposure to zinc-containing nanoparticles. It was observed that increasing the concentration of nanoparticles did not result in a reduction of the total bacterial biomass, which supported the rapid antibacterial activity of these substances. Cytotoxicity was assessed using gingival cells from patients aged 18 to 25 years, and cell viability was determined using the tetrazolium assay. This test assesses mitochondrial function and measures the ability of viable cells to reduce tetrazolium salt to form a purple formazan product. ZnONP was found to be toxic at a concentration of 150 μg/mL, which is higher than the concentration demonstrating significant antibacterial activity.

An analysis of the antibacterial properties of zinc (II)-containing fluorescent coordination polymer ([Zn(DIPT)](NO_3_)(H_2_O)_3_) against *P. gingivalis* [11] was conducted, and growth curves of *P. gingivalis* were generated following exposure to the substance, with measurements of bacterial absorbance taken every 4 h at a wavelength of 630 nm. The optical density decreased from 0.82 to 0.43 after *P. gingivalis* was exposed to the test material, indicating excellent antibacterial activity and the inhibition of *P. gingivalis* growth. One of the studies aimed to prepare hydroxyapatite supplemented with zinc and dextran (ZnHApD) for biomedical applications and analyze its properties [19], assessing the antibacterial activity of ZnHApD samples against *S. aureus*, *P. gingivalis*, and *E. coli* using varying concentrations of ZnHApD for bacterial cultivation. The impact of the ZnHApD solution on the growth of *S. aureus*, *E. coli*, and *P. gingivalis* was evaluated by measuring optical density at 600 nm. The experiments were repeated, and it was observed that *P. gingivalis* was the most susceptible. A reduction in the growth of *P. gingivalis* was noticed after 24 and 48 h at a ZnHApD concentration of 0.075 μg/mL.

In an innovative study, the surface of titanium was modified with zinc, and the biocompatibility and antibacterial properties of this compound were tested [20], including *S. aureus*, *P. gingivalis*, and *A. actinomycetemcomitans* bacteria, as well as three different samples of titanium implants. A titanium SLA implant served as control, while the remaining test titanium implants were rinsed with distilled water, dried in ambient atmosphere, and then coated with zinc. Bacteria were introduced to various titanium samples. The quantities of *S. mutans*, *P. gingivalis*, and *A. actinomycetemcomitans* were significantly reduced on the surfaces of implants coated with zinc. Microscopy revealed distorted membranes of *S. mutans* and *P. gingivalis* located on surfaces covered with zinc, and *A. actinomycetemcomitans* was affected as well. It was demonstrated that zinc had an impact on bacterial cell walls, disrupting their integrity and thus inhibiting their resistance.

The last analyzed study synthesized the coordination polymer {[Zn2(L)2(bdc)]·(H2O)2·DMF}n based on zinc (II) and analyzed its properties [21]. The substance was tested using *P. gingivalis* bacteria, with optical density measured at 600 nm, and growth curves were generated. The results demonstrated that the chemical compound inhibited bacterial growth in vitro. Additionally, the suppressive activity of *P.gingivalis* on the *ragA* and *ragB* genes was observed during the test.

## 4. Discussion

Viability of periodontal tissues depends on the consumption of key nutrients. Microelements such as Zn, Mg, and Cu are vital for the normal metabolism of carbohydrates, lipids, and proteins. Zn is an integral component of antioxidant enzymes and plays a role in the synthesis and activity of hormones closely related to bone metabolism. A decreased amount of zinc may lead to oxidative stress, and its deficiency is associated with poor oral and periodontal health [1]. This substance plays a significant role in immune function, wound healing, protein and DNA synthesis, and cell division [16]. The topical intraoral use of zinc with prolonged release of zinc ions enhances local protection and collagen activity and stimulates wound epithelialization, thereby reducing the risk of infections [2]. In the oral cavity, zinc is found in the saliva, enamel hydroxyapatite, and dental plaque. Local immune activity supports periodontal health and reduces the gingival index [24]. Gingivitis, periodontitis, halitosis, etc. can be successfully treated with zinc supplements.

Zinc is a bivalent cation that is not synthesized in the human body, and therefore, it must be taken as a supplement or obtained from food. The highest amount of zinc is found in wheat, beans, and nuts, while a less amount is found in fruits, vegetables, and tubers. Studies have shown that the utilization of dietary zinc could be markedly different depending on the source of dietary protein. Zinc in diets containing proteins from animal sources (meat, fish, milk, and eggs) has generally been found to be of high availability and well absorbed [25]. Its deficiency is a significant global health problem, especially in developing countries. It is estimated that up to 17% of the world’s population is at risk of zinc deficiency [26]. Zinc deficiency leads to the activation of monocytes/macrophages, which generate free radicals, causing oxidative stress and triggering the production of cytokines such as tumor necrosis factor (TNF-α), interleukin IL-1β, and IL-6 [27]. Zinc deficiency slows wound healing due to decreased activation of the nuclear factor and weakening of the cellular function [2]. Infections, degenerative diseases, damages to the oral mucous membrane, and periodontal tissues are attributed to zinc deficiency [24].

Periodontitis is caused by bacteria accumulating on biofilm covering the teeth; there are more than 700 bacterial species [13]. The main pathogens for periodontal diseases include the Gram-negative bacteria *A. actinomycetemcomitans*, *P. intermedia*, *T. forsythia*, *T. denticola*, and *P. gingivalis* [28,29,30]. These data support the role of red complex pathogens in the etiopathogenesis of periodontal diseases. Bacteria in plaque produce endo- and exotoxins, metabolic products that damage periodontal tissues, penetrate deeper, and cause inflammation and an immune response in macroorganisms [15]. It has been demonstrated that *T. forsythia* (92%), *T. denticola* (82%), *P. intermedia* (70%), *P. gingivalis* (62%), and, less commonly, *A. actinomycetemcomitans* (26%) are associated with moderate periodontitis. All bacterial species (>95%), except for *A. actinomycetemcomitans*, which was found in less than half of the cases, were identified in individuals with severe stages of periodontitis [28,30]. It has been established through a systematic review of the literature [8,9,10,13,14,15,16] that zinc and its compounds have an antimicrobial effect, inhibiting the growth, reproduction, and vital functions of bacteria, and zinc deficiency is frequently associated with diseased states of periodontium [31].

Even small amounts of zinc oxide have strong antimicrobial and bacteriostatic activity. The activity of zinc oxide can be characterized by three modes of action: (1) it generates reactive oxygen species on the bacterial surface; (2) it damages the cellular membrane upon contact; and (3) in an aqueous environment, ZnO releases Zn2+ ions with antimicrobial activity [4]. Zinc peroxide nanoparticles are the key factor in antibacterial activity, especially against anaerobes susceptible to oxygen [13]. It has been proven that antibacterial activity increases with decreasing nanoparticle diameter and increasing concentration; smaller particles adhere more easily to the bacterial membrane or may even penetrate it, which enhances zinc’s activity. Furthermore, zinc peroxide generates electrons, which, upon exposure to ultraviolet or visible light, can interact with water, forming hydroxyl and superoxide anions. The latter can lead to bacterial cell death [14]. Based on the studies, it can be inferred that zinc ions limit bacterial colonization in the gingival sulcus and inhibit plaque formation. Moreover, zinc can depolarize the bacterial membrane, increasing its permeability to hydrogen and susceptibility to acid stress. In some cases, it may result in ruptures of the bacterial membrane, altering its permeability, which is closely related to bacterial sensitivity to the ionic environment. Ion homeostasis affects bacterial reproduction, their interactions, and metabolism. Thus, zinc exhibits inhibitory and detrimental effects on bacteria as it inhibits the glycosyltransferase enzyme, limiting the primary substrate and causing bacterial starvation and death [18].

As in most cases, this scientific literature review also encountered some limitations. The publications examined in vitro studies attempting to simulate the physiological conditions of the human body, but they did not fully correspond to them. As literature analysis indicates that these studies need to be standardized and precisely described [32].

In the studies included in the review, different concentrations of zinc and its compounds were used. The authors applied different research methodologies and analyzed them based on different research analysis and evaluation criteria [8,9,10,11,12,13,14,15,16,17,18,19,20,21]. Unequal ranges of spectrophotometer wavelength were used for the analysis of bacterial concentration based on optical density (from 540 to 660 nm). Several different bacterial species were analyzed in the studies; minimum inhibitory concentration and minimum bactericidal concentration were assessed only in some studies. The significant antimicrobial activity of zinc and its compounds was not demonstrated for all the tested bacteria but only for certain ones. In most cases, the final conclusions assert the impact of these substances on bacterial viability and vital activity changes, specifying and naming specific bacteria. In this case, it would be purposeful to select only those publications where zinc preparations of the same composition were analyzed against a specific type of bacteria. To precisely answer what role zinc plays against a particular periodontal pathogen, a more detailed literature analysis is necessary. By identifying these gaps, we emphasize the directions for future literature analysis.

In vitro cytotoxicity testing of substances is recommended to be conducted according to standardized tests for biological safety assessment, which are formulated as a set of recommendations. This set may be adjusted by modifying many parameters and conditions, provided that the corrections are appropriately justified [32]. Inappropriate conditions can influence the indirect results of in vitro cytotoxicity testing. A detailed description of conditions when studying the cytotoxicity of a substance could facilitate the comparison of different studies [32]. The literature indicates that the cytotoxicity of the substances under investigation is expressed as a percentage of the metabolic activity of the control substance. Substances under investigation for which the activity of the control substance decreased by less than 70% are considered cytotoxic [32]. In this scientific literature analysis, the examination of the biological compatibility and cytotoxic properties of zinc and its compounds does not allow a firm conclusion that zinc preparations for periodontal tissues are entirely safe and noncytotoxic. One limitation of this study is that the selected in vitro studies only investigated the effect of substances on one type of periodontal tissue cells—fibroblasts (the viability of fibroblasts was expressed as a percentage). These cells participate in collagen synthesis, which is an important part of the periodontal structure. However, the studies did not focus on other types of cells that are also part of the structure of periodontal tissues. According to the literature data, exposure and cell type influence the cytotoxicity of the substance under investigation. In this literature review, studies by Dias et al., Mou et al., and Wang et al. were examined, which concluded that zinc-containing substances are not cytotoxic as the survival of the tested fibroblasts ranged from over 60% to 97% [4,8,12]. However, this does not precisely correspond to the data from the previously mentioned literature source. Vergara-Llanos et al. investigated several concentrations of ZnO nanoparticles [10] and demonstrated that a concentration of 50 μg/mL of ZnO nanoparticles was not harmful to fibroblasts as the percentage of surviving cells reached 80%. However, when the concentration was increased to 100 μg/mL, only 50% of viable fibroblasts remained. It was proven that exposure to zinc oxide had a toxic effect depending on the substance’s dose, exposure time, and concentration. As shown by Şeker and Cheng, a concentration of 100 μg/mL also affected structural changes in cells and inhibited fibroblast proliferation [32,33,34].

In periodontology, bacterial plaque is considered one of the main etiological factors of periodontal diseases, and scaling and plaque control are regarded as the primary and effective starting points of treatment. However, it goes without saying that it is impossible to eliminate the bacterial environment. The application of safe doses of topical antimicrobial agents in the treatment of periodontal diseases could enhance disease control and enable the maintenance of healthier periodontal tissues.

## 5. Conclusions

The analysis of the scientific literature confirms the antibacterial action of zinc against periodontal pathogenic bacteria. Zinc and its compounds suppress the vital functions, growth, and reproduction of microorganisms such as *A. actinomycetemcomitans*, *P. intermedia*, *T. denticola*, *T. forsythia*, and *P. gingivalis*, disrupting the integrity of bacterial cell walls. This finding supports the idea that the properties of zinc have the potential to contribute to the maintenance of healthier periodontal tissues. At low concentrations, these substances do not exhibit cytotoxic effects on fibroblasts. Considering these results, it could be argued that zinc and its compounds could be included in pharmaceutical preparations intended for clinical use in periodontology.

## Figures and Tables

**Table 1:** Flow chart of the study: database selection, keyword formulation, and publication analysis.

Data Base	Keyword Formulation	Number of Publications (with Filters Applied/Number of Selected Publications)
Web of Science	Zinc AND *Porphyromonas gingivalis*	69/25
	Zinc AND *Tannerella forsythia*	7/3
	Zinc AND *Treponema denticola*	7/2
	Zinc AND *Prevotella intermedia*	10/5
	Zinc AND *Aggregatibacter actinomycetemcomitans*	12/4
PubMed	Zinc AND *Porphyromonas gingivalis*	52/16
	Zinc AND *Tannerella forsythia*	6/2
	Zinc AND *Treponema denticola*	6/2
	Zinc AND *Prevotella intermedia*	10/5
	Zinc AND *Aggregatibacter actinomycetemcomitans*	15/3
ScienceDirect	Zinc AND *Porphyromonas gingivalis*	205/7
	Zinc AND *Tannerella forsythia*	23/4
	Zinc AND *Treponema denticola*	31/4
	Zinc AND *Prevotella intermedia*	50/4
	Zinc AND *Aggregatibacter actinomycetemcomitans*	55/4

**Table 2:** General characteristics of scientific publications.

No.	Author, Year and Reference No.	Bacteria Examined (Amount)	Test Substance	Incubation Conditions and Duration	Results
1	Dias et al., 2018 [4]	*P. gingivalis, P. intermedia,* and *A. actinomycete mcomitans* (100 µL)	ZnO	Anaerobic environment (90% nitrogen + 10% CO_2_); 37 °C, 24 h	Weak antibacterial activity, inhibition zone of 6.5 mm.
			ZnO + PCL		No antibacterial activity, no inhibition zone formed.
2	Wang et al., 2019 [8]	*P. gingivalis* (100 µL)	ZnO nanoparticles	Anaerobic environment 37 °C, 48 h	Antibacterial activity distinguished at 40 µg/mL concentration of test substance, inhibition zone of 18.09 mm.
3	Mou et al., 2019 [12]	*P. gingivalis* (100 µL)	ZnO + albumin nanoparticles	Anaerobic environment 37 °C, 24 h	Antibacterial activity distinguished, inhibition zone of 13.36 mm.
		*P. intermedia* (100 µL)			Antibacterial activity distinguished, inhibition zone of 14.01 mm.
4	Fröber et al., 2019 [13]	*P. gingivalis* (-)	ZnO + glucose-1- phosphate	Anaerobic environment (7.5–8% CO_2_) 37 °C, 24–48 h	Antibacterial activity distinguished at 100 µg/mL concentration of test substance.
		*P. intermedia* (-)			Antibacterial activity distinguished at 25 µg/mL concentration of test substance.
		*A. actinomycete* *mcomitans* (-)			Antibacterial activity distinguished at 50 µg/mL concentration of test substance.
5	Bergs et al., 2017 [14]	*P. gingivalis* (-)	ZnO + glucose-1- phosphate	Anaerobic environment (5–10% CO_2_) 37 °C, 24–48 h	Antibacterial activity distinguished at 100 µg/mL concentration of test substance.
		*P. intermedia* (-)			Antibacterial activity distinguished at 20 µg/mL concentration of test substance.
		*A. actinomycete mcomitans* (-)			Antibacterial activity distinguished at 20 µg/mL concentration of test substance.
6	Vargas-Reus et al., 2012 [16]	*P. gingivalis* (100 µL)	ZnO	Anaerobic environment, 48 h	Antibacterial activity distinguished at 250 µg/mL concentration of test substance.
		*P. intermedia* (100 µL)			Antibacterial activity distinguished at 100 µg/mL concentration of test substance.
		*A. actinomycete mcomitans* (100 µL)			Antibacterial activity distinguished at 250 µg/mL concentration of test substance.
7	Vergara-Llanos et al., 2020 [10]	*P. gingivalis* (-)	ZnO nanoparticles	Anaerobic environment, 37 °C, 24 h	Antibacterial activity distinguished at 78.3 µg/mL concentration of test substance.
8	Pizzey et al., 2011 [9]	*P. gingivalis* (-)	Zn gluconate	Anaerobic environment, 37 °C, 24–48 val.	Antibacterial activity distinguished at 2.76 mM concentration of test substance
9	Kang et al., 2017 [15]	*P. gingivalis* (150 µL)	ZnCl_2_	Anaerobic environment 37 °C, 4–30 h	Antibacterial activity distinguished at 0.0625% concentration of test substance.
		*P. intermedia* (150 µL)			Antibacterial activity distinguished at 0.0625% concentration of test substance.
		*T.forsythia* (150 µL)			Antibacterial activity distinguished at 0.125% concentration of test substance.
		*T.denticola* (150 µL)			Antibacterial activity distinguished at 0.0625% concentration of test substance.
		*A. actinomycete* *mcomitans* (150 µL)			Antibacterial activity distinguished at 0.25% concentration of test substance.
10	Zhao et al., 2020 [17]	*P. gingivalis* (-)	[Zn(TBTA) (L)1.5]n	Anaerobic environment (10% CO_2_); 37 °C, 24 h	Antibacterial activity distinguished according to the decline of the growth curve.
11	Wang et al., 2019 [21]	*P. gingivalis* (100 µL)	Zn(NO_3_)_2_·6H_2_O	Anaerobic environment (80% N_2_, 10% H_2_, and 10% CO_2_), 24 h	Antibacterial activity distinguished according to the decline of the growth curve.
12	Niu et al., 2020 [11]	*P. gingivalis* (-)	Zn(NO_3_)_2_·6H_2_O	Anaerobic environment (80% N_2_, 10% H_2_, and 10% CO_2_), 24 h	Antibacterial activity distinguished by decrease in optical density from 0.82 up to 0.43 units of measurement.
13	Predoi et al., 2019 [19]	*P. gingivalis* (-)	ZnHApD	Anaerobic environment, 37 °C, 24–48 h	Antibacterial activity distinguished by decrease in optical density.
14	Shao et al., 2019 [20]	*P. gingivalis* and *A. actinomycete mcomitans* (-)	Zinc-coated titanium	37 °C, 2 days	Antibacterial activity distinguished as no viable bacteria were detected on the surface.
15	Toledano-Osorio et al., 2018 [18]	*P. gingivalis* (-)	Zn nanoparticles	Anaerobic environment, 37 °C, 3–24 h	Antibacterial activity distinguished by 93% decrease in bacteria count.

“(-)”—no data presented; ZnO—zinc oxide; [Zn(TBTA) (L)1.5]n—bivalent zinc supporting polymer; ZnCl—zinc chloride; Zn(NO_3_)_2_·6H_2_O—bivalent zinc supporting polymer; ZnHApD—dextran-coated zinc hydroxyapatite; CO_2_—carbon dioxide.

**Table 3:** Antibacterial activity according to bacterial inhibition.

No.	Author, Year, and Reference No.	Test Period (h)	Inhibition Zone (mm)						Results
			P.g	P.i	T.f	T.d	A.a	For the Bacteria Studied Together	
1	Dias et al., 2018 [4]	24	-	-	-	-	-	6.5 (SD 0.5)	Antibacterial activity distinguished
2	Wang et al., 2019 [8]	24	18.09	-	-	-	-	-	Antibacterial activity distinguished, *p* < 0.001
3	Mou et al., 2019 [12]	24	13.36	14.01	-	-	-	-	Antibacterial activity distinguished, *p* < 0.01

P.g—Porphyromonas gingivalis; P.i—Prevotela intermedia; T.f—tannerella forsythia; T.d—Treponema denticola; A.a—Aggregatibacter actinomycetemcomitans; “-“—no information presented.

**Table 4:** Estimated minimum inhibitory concentration (MIC) and minimum bactericidal concentration (MBC).

No.	Author, Year and Reference No.	Test Period (h)	MIC/MBC (µg/mL)						Results
			P.g	P.i	T.f	T.d	A.a	For the Bacteria Studied Together	
1	Wang et al., 2019 [8]	24	40/40	-	-	-	-	-	
2	Vergara-Llanos et al., 2020 [10]	24	-	-	-	-	-	78.3–3906	
3	Frober et al., 2019 [13]	24	100/100	25/25	-	-	50/50	-	
4	Bergs et al., 2017 [14]	24	100/100	20/100	-	-	20/100	-	
5	Vargas-Reus et al., 2012 [16]	48	250/250	100/100	-	-	250/250	-	*p* < 0.05

P.g—Porphyromonas gingivalis; P.i—Prevotela intermedia; T.f—tannerella forsythia; T.d—Treponema denticola; A.a—Aggregatibacter actinomycetemcomitans; “-“—no information presented.

## Data Availability

Not applicable.
